# Predictors of elevated sperm DNA fragmentation: a morphology-based approach to semen analysis

**DOI:** 10.1186/s12610-025-00297-8

**Published:** 2025-12-15

**Authors:** Mahsa Kazemi, Ali Moradi, Fatemeh Bayat, Saghar Salehpour, Sarah Niakan, Hamid Nazarian

**Affiliations:** 1 Department of Biology and Anatomical Sciences, School of Medicine, Shahid Beheshti University of Medical Sciences, Tehran, Iran; 2 Infertility and IVF Center, Taleghani Hospital, Shahid Beheshti University of Medical Sciences, Tehran, Iran; 3 Department of Obstetrics and Gynecology, Preventive Gynecology Research Center, Shahid Beheshti University of Medical Sciences, Tehran, Iran; 4 Department of Obstetrics and Gynecology, Faculty of Medicine, Shahed University, Tehran, Iran

**Keywords:** Male infertility, Semen analysis, Sperm morphology, Sperm DNA integrity, Infertilité masculine, Analyse du Sperme, Morphologie des Spermatozoïdes, Intégrité de l’ADN des Spermatozoïdes

## Abstract

**Background:**

Empirical evidence indicates that high levels of sperm DNA fragmentation (SDF) negatively impact the results of both natural conception and assisted reproductive technology (ART). However, there is a notable absence of detailed guidelines for clinicians on which patient groups should be tested for SDF based on their semen analysis results. The goal of this study was to determine which sperm categorizations and morphological subcategorizations should be tested for sperm DNA integrity.

**Results:**

Lowered sperm concentration, motility, progressive motility, and morphology, as well as a higher percentage of immature sperm, were linked to increased DNA Fragmentation Index (DFI) values. Higher DFI values were also found in semen samples from patients with two-sided varicoceles. The most significant intermediate correlation was identified between DFI and micro/partial head defects. Additionally, a low but significant correlation was observed in cases of combined oligo-astheno-teratozoospermia.

**Conclusion:**

Targeted DFI assessment is especially valuable for patients who have a two-sided varicocele, micro/partial head sperm defects, or combined oligo-astheno-teratozoospermia. Incorporating this assessment may enhance the diagnostic accuracy of male infertility evaluations.

## Résumé

**Contexte:**

Les preuves empiriques indiquent que des niveaux élevés de fragmentation de l’ADN spermatique ont un impact négatif sur les résultats à la fois de la conception naturelle et des techniques de procréation médicale assistée. Cependant, il existe une absence notable de directives détaillées pour les cliniciens quant aux groupes de patients à tester pour la fragmentation de l’ADN spermatique en fonction de leurs résultats d’analyse sperme. L’objectif de cette étude était de déterminer quelles catégorisations et sous-catégorisations morphologiques des spermatozoïdes devaient être testées pour l’intégrité de l’ADN des spermatozoïdes.

**Résultats:**

Une diminution de la concentration, de la motilité, de la motilité progressive et de la morphologie des spermatozoïdes, ainsi qu’un pourcentage plus élevé de spermatozoïdes immatures, ont été associés à une augmentation de l’indice de fragmentation de l’ADN (DFI). Des valeurs plus élevées de DFI ont également été observées dans des échantillons de sperme chez des patients atteints de varicocèles bilatérales. La corrélation intermédiaire la plus significative a été identifiée entre DFI et anomalies micro/partielles de la tête des spermatozoïdes. En outre, une corrélation faible mais significative a été observée dans les cas d’oligoasthénotératozoospermie combinée.

**Conclusion:**

L’évaluation ciblée par l’indice de fragmentation de l’ADN (DFI) est particulièrement précieuse pour les patients présentant une varicocèle bilatérale, des malformations micro/partielles de la tête des spermatozoïdes, ou une combinaison d’oligoasthénotératozoospermie. L’intégration de cette évaluation pourrait améliorer la précision diagnostique des évaluations de l’infertilité masculine.

## Introduction

Infertility represents a global health concern, and various nations’ population policies are endeavoring to advance the diagnosis and treatment of this widespread issue. Infertility affects approximately 17% of individuals during their reproductive lifespan, imposing a significant global health burden [1] and contributing to nearly 50% of infertile couples, either as the sole cause or in combination with female factors [2]. One of the fundamental challenges in male infertility is the accurate evaluation of sperm quality and its effect on the success of assisted reproductive technologies (ART). Although conventional semen analyses, including sperm count, motility, and morphology, provide valuable insights, many men with normal results in these tests still face infertility issues. This discrepancy highlights the need to investigate more specific parameters, such as sperm DNA integrity (DNA Fragmentation Index (DFI)), which has been shown to influence embryonic development, blastocyst quality, and even the long-term health of offspring and has emerged as a valuable predictor of assisted reproductive technology (ART) outcomes [3–6].

Although DFI is a crucial tool for measuring sperm DNA integrity, it often represents an additional cost to patients [5]. However, if DFI is not assessed, the patient may not receive appropriate treatment, potentially leading to unsuccessful fertility treatments. Despite this evidence, there is a paucity of clear guidelines that delineate which patients should undergo DFI measurements based on seminal profiles. The present study aimed to bridge this gap by evaluating the associations between standard semen classifications, detailed sperm morphology subcategories, and DFI, with the ultimate goal of informing clinical decision-making for male infertility assessment.

## Patients and methods

### Study design

**Fig. 1:**
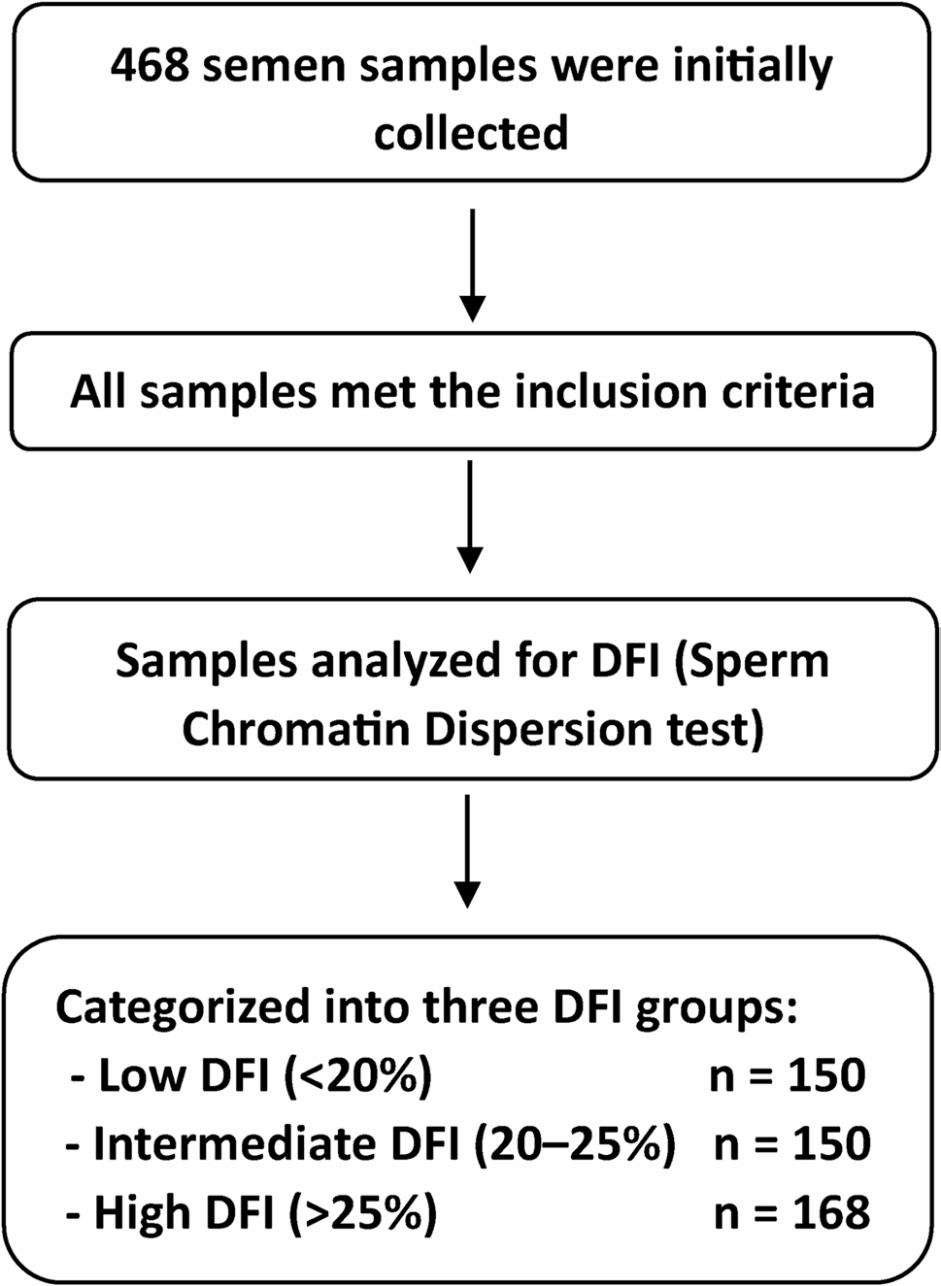
Flow diagram of sample inclusion and grouping according to sperm DNA fragmentation index (DFI). A total of 468 semen samples were collected. All samples met the inclusion criteria and were categorized into three DFI groups: low (<20%, n = 150), intermediate (20–25%, n = 150), and high (>25%, n = 168). All 468 samples were included in the final statistical analysis

In this cross-sectional study, semen samples from 468 men aged 20–55 years who were referred to the infertility center of Taleghani Hospital, Tehran, Iran, were obtained through masturbation after 3–5 days of abstinence (Fig. 1). Figure 1 illustrates the overall flow of participant inclusion in the study.

After liquefaction, a standard semen analysis was conducted manually according to the WHO guidelines (2021) [7].

### Sperm morphology assessment

Abnormal sperm morphology was assessed under bright-field microscopy using a Nikon Eclipse microscope equipped with a high-resolution Nikon 12-bit CCD (Nikon DS-Q) and a 40×fluorite objective.

Semen smears were air-dried and stained using Diff-Quik (IVF Co., Iran). All samples were analyzed using 100× immersion oil objectives, and 200 spermatozoa were scored. Each semen sample was classified based on its morphological characteristics.

Only one morphological variation was registered for each spermatozoon, in the following order of priority: (i) head defects: large, pyriform, tapered, Pin, Micro/Partial Head, globozoospermia, amorphous head shape, vacuolated (> 2 vacuoles or > 20 of the head area occupied by vacuolar areas), vacuoles in the post-acrosomal region, small or large acrosomal areas (< 40% or > 70% of the head area), and double heads; (ii) neck defects: bent, asymmetrical insertion, thick or irregular, abnormally thin, and excess residual cytoplasm (one-third or more of the sperm head size); (iii) tail defects: multiple, short, stump, and coiled. This means that a spermatozoon showing head, neck, or head and tail defects was classified according to its head defect only. Morphological evaluation of each spermatozoon was performed using the Sperm Chromatin Dispersion test (SCD)and SCMA assays.

### Sperm DNA integrity assessment

The Sperm Chromatin Dispersion test (SCD; IVF Co., Iran) was conducted to analyze the DFI according to the manufacturer’s instructions. This assay relies on measuring the dispersion of nuclear chromatin following the removal of nuclear proteins and the denaturation of DNA. In sperm with fragmented DNA, the outer halo of the dispersed DNA loop is not evident, while it is distinctly visible in normal sperm with intact DNA. Based on this understanding, 300 spermatozoa from each sample were examined at 1000 × magnification using a bright-field microscope. The percentage of sperm with fragmented DNA was subsequently documented. Based on the measured sperm DNA fragmentation index, participants were categorized into three groups: low DFI (< 20%), intermediate DFI (20–25%), and high DFI (> 25%)(Fig. 1).

### Sperm chromatin maturation assessment

The assessment of sperm chromatin maturation was conducted using an aniline blue (AB) staining technique. Each sample’s fresh sperm smear was first air-dried, then fixed, and subsequently stained with the SCMA (Sperm Chromatin Maturation Assay) kit (IVF Co., Iran), according to the manufacturer’s instructions. Following this, 200 spermatozoa from each stained smear were examined under oil immersion at a magnification of 1000× using a bright-field microscope. The percentage of sperm displaying immature chromatin, identified by blue-stained nuclei, was then recorded.

### Reproducibility assessment

All slides (morphology, SCD and SCMA) were assessed by a single experienced embryologist who was blinded to the clinical data. To evaluate intra- and inter-observer reproducibility, a random subset of 29 slides was re-scored by the same observer after an interval of ≥ 2 weeks and independently by a second trained observer. Agreement for continuous measures (DFI) was quantified using the intraclass correlation coefficient (ICC). Agreement for categorical classification (DFI ≥ 25% vs. < 25%) was assessed using Cohen’s kappa. Analytical variability was further summarized by the mean absolute percent difference between paired scores.

### Ethical considerations

The Research Ethics Committees of the Faculty of Medicine, Shahed University, Tehran, Iran, approved this study (Code: IR.SHAHED.MED.REC.1404.031).

Informed written consent was obtained from all individual participants included in the study.

### Statistical analysis

Statistical analyses were performed using the Statistical Package for the Social Sciences (version 20.0; SPSS Inc., Chicago, Illinois, USA). Normality was tested using the Shapiro–Wilk test. Group comparisons were conducted using ANOVA and Kruskal-Wallis tests, as appropriate, followed by Tukey’s post-hoc test. Correlations between morphology and DFI were analyzed using Pearson’s correlation, with significance set at *P* < 0.05.

## Result

168 samples from 468 men were included in the High DFI group, and 2 groups of 150 samples were included in either the low or intermediate DFI group (Fig. 1).

### Reproducibility results

Reproducibility was assessed on a random subset of 29 slides. Intra-observer agreement for DFI (baseline vs. re-score by the same observer) was excellent (ICC = 0.968). Inter-observer agreement (baseline vs. second observer) was also excellent (ICC = 0.994), and ICC between the two observers was 0.960. For the categorical classification using a 25% DFI threshold (fragmented vs. non-fragmented), Cohen’s kappa values were 0.84 (baseline vs. observer1), 1.00 (baseline vs. observer2), and 0.84 (observer1 vs. observer2), indicating substantial to almost-perfect agreement. The mean absolute percent difference between paired scores was 11.08% (baseline vs. observer1), 4.52% (baseline vs. observer2), and 11.35% (observer1 vs. observer2).

The demographic and clinical characteristics of the study participants were stratified by DFI levels (< 20%, 20–25%, and > 25%) and are summarized in Table 1. The mean age and infertility duration were comparable across groups with low, intermediate, and high DFI values (*p* > 0.05). However, the prevalence of bilateral varicocele was significantly higher in men with high DFI (*p* < 0.01). No differences were observed regarding other factors such as smoking.

**Table 1:** Demographic and clinical characteristics of participants according to sperm DNA fragmentation index (DFI) levels (< 20%, 20–25%, and > 25%)

			**DFI**			*P value*
			**<20**	**20-25**	**>25**	
Age (yr)			38.80±4.52	36.6±5.91	35.72±4.62	≥0.05
Infertility Duration (yr)			3.526±1.86	2.90±1.19	3.32±1.37	≥0.05
Semen Categorization	Normozospermia (9.79%) n=46		2.7	1.91	5.18	≥0.05
	Teratozoospermia (22.2%) n=104		6.8	7.1	8.3	≥0.05
	Oligo-teratozoospermia (17.2%) n=80		2.3a	3.7a	11.2b	**0.01**
	Astheno-teratozoospermia (29.9%) n=140		6.8a	6.2a	16.9b	**0.01**
	oligo-astheno teratozoospermia (20.91%) n=98		1.8a	3.1a	16.01b	**0.001**
Varicocele	Yes	One-sided (15.5%) n=72	7.8	3.3	4.4	≥0.05 **0.05**
		Two-sided (15.4%) n=72	3.3a	3.3a	8.8b	
	No (69%) n=323		17.8	25.6	26.7	≥0.05
Smoking	Yes (39.9%) n=187		12.2	15.6	12.1	≥0.05
	No (60.1%) n=281		16.7	16.7	26.7	≥0.05

This table presents demographic data such as age and infertility duration, along with semen categorization, varicocele presence, and smoking status, all broken down by DNA Fragmentation Index (DFI) categories (<20 (n=150), 20–25 (n=150), >25 (n=168)). The legend specifies that the data is presented as Mean ± SD and that statistically significant data (P-value < 0.05) are shown in bold. Different superscript letters (a, b) indicate significant differences between groups according to Tukey’s post-hoc test (p < 0.05)

As shown in Table 2, the comparison of conventional semen parameters according to DFI categories, men with high DFI demonstrated markedly lower sperm concentrations than those with low and intermediate DFI, with a statistically significant difference (*p* = 0.005). Progressive and total motility were also significantly reduced in the high DFI group (*p* < 0.01). and the percentage of immature sperm was associated with increased DFI values. Morphology followed a similar declining trend at higher DFI. In contrast, semen volume did not differ significantly between the groups.

**Table 2:** The comparison of conventional semen parameters according to DFI category

Parameters	DFI			*P* value
	< 20	20–25	> 25	
Concentration	51.28 ± 13.45^a^[49.10, 53.46]	58.74 ± 12.01^a^[56.80, 60.68]	39.07 ± 8.42^b^[37.78, 40.36]	**0.005**
Progressive motility	36.19 ± 8.57^a^[34.80, 37.58]	35.90 ± 10.59^a^[34.20, 37.60]	26.16 ± 5.48^b^[25.33, 26.99]	**0.01**
Total motility	58.54 ± 12.83^a^[56.46, 60.62]	55.22 ± 13.28^a^[53.08, 57.36]	46.65 ± 8.85^b^[45.30, 48.00]	**0.02**
Morphology	0.82 ± 1.33^a^[0.60, 1.04]	0.73 ± 1.07^a^[0.55, 0.91]	0.42 ± 0.79^b^[0.30, 0.54]	**0.01**
SCMA	20.21 ± 3.77^a^[19.60, 20.82]	27.20 ± 3.52^ab^[26.63, 27.77]	36.19 ± 2.04^b^[35.87, 36.51	**0.01**

This table shows the relationship between semen parameters (concentration, motility, morphology, and SCMA) and the DFI categories (<20 (n=150), 20–25 (n=150), >25 (n=168)). The legend indicates that the data is presented as mean ± SD with a 95% confidence interval (CI) shown in brackets. Comparisons were made using one-way ANOVA with Tukey’s HSD for post-hoc tests

*Abbreviations*:* DFI* DNA Fragmentation Index, *SCMA* Sperm Chromatin Maturation

^*^Sperm chromatin maturation assay (immature index)

While the percentage of immaturity in the DFI categorization showed an increasing trend, the sub-categorized morphological defects did not fulfill the significance criterion despite micro/partial head abnormality. Considering 24% as the immaturity cut-off, the percentage of tapered head sperm was higher in immature sperm with higher DFI compared to more normal ones (13.12 ± 1.37 and12.50 ± 2.72 versus 9.69 ± 1.08, p-value 0.07).

DFI and semen categorization showed a significantly low power correlation (correlation coefficient 0.26, p-value 0.05) between DFI and asthenozoospermia combined with oligozoospermia or teratozoospermia.

The distribution of sperm morphological subcategories and their correlation with DFI are illustrated in Table 3. Among the various head, neck, and tail defects, micro- and partial-head abnormalities showed the strongest positive correlation with DFI (*r* = 0.40, *p* < 0.01). Other head defects, such as tapered or amorphous forms, also tended to increase with higher DFI, although not all of them reached statistical significance.

**Table 3:** Morphological sub-abnormalities in DFI-categorized samples

Morphology Characteristics (%)		DFI			*P* value
		< 20	20–25	> 25	
Head Abnormalities	Tapered	9.69 ± 1.08	12.50 ± 2.72	13.12 ± 1.37	≥ 0.05
	Pin	7.10 ± 1.90	6.76 ± 1.20	6.60 ± 1.08	≥ 0.05
	Piriform	11.13 ± 2.96	9.88 ± 2.21	7.50 ± 1.83	≥ 0.05
	Big	8.82 ± 1.93	11.96 ± 2.01	8.69 ± 0.56	≥ 0.05
	Vacuolar	14.14 ± 1.0	18.36 ± 2.24	9.46 ± 2.60	≥ 0.05
	Double Head or Tail	3.00 ± 0.55	3.00 ± 1.41	5.33 ± 1.51	≥ 0.05
	Globozospermia	7.00 ± 1.33	8.00 ± 1.11	8.13 ± 2.31	≥ 0.05
	Micro/Partial Head	12.73 ± 1.36^a^	14.55 **±** 2.15^a^	26.93 **±** 1.09^b^	**0.01**
	Abnormal	37.46 ± 1.91	33.11 ± 1.52	35.33 ± 1.58	≥ 0.05
Neck Abnormality	Swelled	8.95 ± 2.16	7.00 ± 1.53	8.11 ± 1.47	≥ 0.05
Tail Abnormalities	Coiled	4.78 ± 0.20	6.08 ± 1.99	7.59 ± 1.19	≥ 0.05
	No tail	2.36 ± 1.74	3.54 ± 0.66	2.42 ± 1.83	≥ 0.05
	Stump	5.40 ± 1.50	4.15 ± 0.86	3.36 ± 1.05	≥ 0.05

This table details the percentages of various sperm morphological sub-abnormalities (head, neck, and tail defects) across the different DFI categories (< 20 (*n* = 150), 20–25 (*n* = 150), > 25 (*n* = 168)). The explanatory text notes that the data are presented as mean ± SD and defines DFI. It also specifies the statistical methods used (one-way ANOVA with Tukey’s HSD post-hoc tests). It highlights that only micro/partial head abnormality showed a significant correlation with DFI (*r* = 0.40, *p* = 0.008)

*Abbreviations*: *DFI* DNA Fragmentation Index

Overall, our findings reveal that increasing sperm DNA fragmentation is associated not only with poorer semen parameters but also with specific sperm head abnormalities.

## Discussion

Assessment of sperm DNA integrity is important in the evaluation of male fertility potential. However, given that this test may not be necessary for every couple who visits an infertility center and may impose additional costs on them, it is essential to determine which couples’ DFI testing is beneficial. Therefore, in this study, we evaluated the relationship between sperm categorization and percentage of sperm DNA fragmentation by examining semen samples from couples visiting an infertility center. To study which type of sperm morphology is related to sperm DNA fragmentation, we examined the relationship between several important morphological subcategorizations and DFI.

According to the findings of this study, no significant difference was observed in the groups with low, medium, and high DFI in normal sperm samples (normozoospermia). DFI and semen categorization showed a significantly low power correlation (correlation coefficient 0.26, p-value 0.05) between DFI and asthenozoospermia combined with oligozoospermia or teratozoospermia. Research indicates that normozoospermic patients exhibit lower DFI levels than those with altered parameters, including oligozoospermia, asthenozoospermia, teratozoospermia, and oligoasthenoteratozoospermia [8]. Additionally, previous research found that patients with abnormal sperm parameters had lower antioxidant levels and higher oxidative stress markers in seminal plasma than normozoospermic men [9]. These findings suggest that impaired sperm parameters are associated with increased DNA fragmentation and oxidative stress, potentially contributing to male infertility [10].

According to our results, semen characteristics in DFI categories showed that in the group with high DFI, sperm count, total sperm motility, progressive motility, and sperm morphology were significantly lower than in the groups with medium DFI, which proves that sperm disorders in semen analysis can be associated with sperm integrity disorders. Previous research has shown a negative correlation between DFI and progressive motility as well as normal morphology [8]. Ferrigno et al. (2021) found a significant correlation between abnormal sperm morphology and DNA damage [11]. These findings suggest that impaired semen parameters are associated with increased DNA fragmentation, which may affect fertility potential and assisted reproductive technique outcomes.

Our study findings showed that sperm maturity significantly declined in the group with a high DFI. Studies have demonstrated that Abnormal chromatin condensation and protamine deficiency are associated with increased DNA damage and decreased sperm quality [12]. Furthermore, sperm protamine content and DNA damage are closely associated, with protamine deficiency likely contributing to DNA instability and damage, potentially affecting fertility [13].

Research has indicated a significant association between varicocele and sperm DNA damage [14, 15]. As shown in this study, varicocele on both sides was associated with a higher DFI. Patients with varicocele exhibit higher levels of DNA fragmentation than the controls [14]. This DNA damage is observed in both normozoospermic and oligoasthenoteratozoospermic men with varicoceles [16]. Varicocele patients also show higher rates of chromatin abnormalities and immature sperm than fertile men [17]. Importantly, varicocelectomy can improve sperm DNA integrity, with studies reporting significant decreases in DNA fragmentation indices and improvements in chromatin compaction post-surgery [14, 18]. The pathogenesis involves an imbalance between excessive reactive oxygen species (ROS) production and antioxidant protection, leading to sperm DNA damage [19]. Oxidative stress correlates positively with DNA fragmentation and chromatin decondensation in patients with varicocele [20].

Sperm morphology, a critical indicator of structural and functional health, is widely used in fertility assessments. Morphological features often reflect underlying chromatin packaging defects or oxidative stress during spermatogenesis, both of which can lead to DNA fragmentation (5–7). To study which morphology abnormality sub-categorization can be associated with DFI-categorization, Table 3 was used. DFI and sub-categorized morphology indicated a significant intermediate power correlation (correlation coefficient 0.4, *p* = 0.008) between DFI and sperm morphology with micro/partial heads (Table 3). Research has revealed a complex relationship between sperm morphology and DNA integrity. Similar to our findings, some studies have reported a significant correlation between normal sperm morphology and DNA integrity [12, 21]. Abnormal sperm morphology, particularly head defects, is associated with higher DFI values [11, 18]. Specifically, round-headed spermatozoa and amorphous heads are more prevalent in samples with a higher DFI [22]. Quantitative analysis of sperm head shape revealed that abnormal ellipticity and angularity were significantly correlated with DNA fragmentation [23]. Additionally, the presence of large nuclear vacuoles in sperm heads is associated with increased DNA fragmentation [23]. Interestingly, our findings showed that micro/partial head sperm samples had higher DFI values. However, others have reported that morphology alone is insufficient to predict DNA fragmentation [22, 24]. It is of note how this structural abnormality can appear under oxidative stress, and whether it can be rehabilitated by antioxidant therapy. No correlation was detected between the specific morphological abnormalities and semen categorization. Previous studies have revealed that abnormal sperm head shapes, such as tapered or elongated heads, are associated with higher rates of protamine deficiency and DNA fragmentation [25]. These findings suggest that sperm morphology can indicate underlying dysfunction and highlight the importance of considering sperm head morphology in assessing DNA integrity and potential fertility outcomes, particularly in assisted reproductive techniques, such as ICSI [11].

Notably, the relationship between morphology and DNA damage is complex [26].

Several mechanisms have been proposed to explain the association between sperm head morphology and DNA integrity.

Morphogenesis failure: This can lead to sperm structural defects, diminished sperm maturity, and sperm nuclear DNA fragmentation (SDF) due to impaired repair of nuclear DNA strand breaks during the early spermatid stage [27].

- II. Oxidative stress: Sperm with diminished maturity are known to generate higher levels of reactive oxygen species, which can increase DNA fragmentation [27].
- III. Chromatin condensation: The shaping of sperm heads may depend on specific patterns of DNA-protein complex assembly established during chromatin condensation in the nuclei [28]. Abnormalities in this process can affect both morphology and DNA integrity.
- IV. Spermiogenesis: During this phase, significant rearrangements, including chromatin condensation and nuclear reshaping, occur in the sperm nucleus. Impairment of this process may lead to morphological abnormalities and DNA damage [29].

We acknowledge that the observed correlations between DFI and clinical features such as asthenozoospermia, micro/partial head defects and bilateral varicocele were of low-to-moderate strength (e.g. r values in the range reported above) and therefore should be interpreted as associations rather than definitive predictive thresholds. Larger prospective studies are required to determine whether specific DFI cut-offs can be validated for clinical decision-making.

Limitations of the Study are the small sample size, which could affect the generalizability of the findings. The document also notes that the methods for SDF assessment (Sperm Chromatin Dispersion test) may not be considered the absolute gold standard in all clinical settings, which could be another point of consideration.

## Conclusion

The results of this study recommend the use of DNA integrity assessment in patients with asthenozoospermia sperm category, micro/partial head defects in sperm analysis, and two-sided varicocele in fertility assessment. These findings suggest that DNA integrity testing could be particularly beneficial for specific subgroups of infertile men, potentially improving the diagnostic accuracy and treatment planning. Implementing this recommendation may lead to more targeted interventions and better outcomes in couples struggling with male factor infertility. Furthermore, incorporating DNA integrity assessment into routine fertility evaluations for these identified patient groups could enhance the overall effectiveness of assisted reproductive technologies.

## Data Availability

The data and materials used in this study are available from the corresponding author upon reasonable request.

## Abbreviations

SDF: Sperm DNA Fragmentation
ART: Assisted Reproductive Technology
DFI: DNA Fragmentation Index
WHO: World Health Organization
SCD: Sperm Chromatin Dispersion
SCMA: Sperm Chromatin Maturation Assay
ROS: Reactive Oxygen Species
ICSI: Intra-cytoplasmic sperm injection
